# Fingertip Necrosis due to Intravascular Use of Mephedrone: A Case Report

**DOI:** 10.1097/GOX.0000000000001906

**Published:** 2018-08-07

**Authors:** Mónica Francés, Víctor Fuertes, José M. Casarrubios, Carlos Navarro, Olivia C. Sánchez, Estefania Poza, Javier Fernández-Palacios

**Affiliations:** From the University Hospital of Gran Canaria Dr. Negrín - Plastic and Reconstructive Surgery Department. Las Palmas de Gran Canaria, Spain.

## Abstract

Mephedrone is a new synthetic cathinone-derived drug. It is a sympathomimetic drug, and its structure is similar to amphetamines, although its specific pharmacokinetics and metabolism remain unknown. We performed a literature search in PUBMED with the following key words: Mephedrone AND Necrosis AND Hand. No results were found. We performed a second literature search with the following key words: Mephedrone AND Physiopathology AND Side effects, obtaining a total of 7 articles that we read before writing this case report. We will present a case report of a 28-year-old man with distal ischemia in his left hand associated to intra-arterial drug use of mephedrone. The patient ended up having superficial necrosis involving skin and subcutaneous tissue in his thumb, which was treated with wound care. Good quality healing, full range of motion, and normal sensitivity were achieved. There are no previous publications related to any side effects secondary to its intravascular use. The ultimate mechanism producing this distal fingertip necrosis remains undefined.

## BACKGROUND

Mephedrone is a synthetic cathinone-derived drug from Khat, a plant whose leaves are consumed because of its stimulating properties in East Africa. It is colloquially known as “bath salts,” “Miau,” “MMCAT.”^1^

Its first use as a recreational drug was reported in 2008. Despite of the efforts to regulate its trade, there has been an increase in its popularity, because of its broad availability and the lack of a specific urine test to detect it.

It is mostly consumed as a snort drug. Intravascular/intramuscular administration is less common. Only 7% of its users consume it as single drug, whereas 93% do it in combination with other substances.

Mephedrone has a sympathomimetic effect increasing extracellular levels of aminic neurotransmitters^2^ working as a reuptake inhibitor. Its metabolism and pharmacokinetics remain unknown. There have been 2 studies performed in rats and humans,^3,4^ revealing that its metabolism is similar to amphetamines and that it has urine excretion.

The clinical effects can overlap to the ones produced by cocaine, methamphetamine, or Methylenedioxymethamphetamine (MDMA).

Its most common side effects reported in the United Kingdom Toxicology records are cardiologic and neuropsychiatric events.^5^

## METHODS

The aim of this article is to show a clinical case of a distal digital necrosis after intravascular administration of mephedrone for recreational use. We performed a literature search in PUBMED with the following key words: Mephedrone AND Necrosis AND Hand. No results were found. We performed a second literature search with the following key words: Mephedrone AND Physiopathology AND Side effects, obtaining a total of 7 articles. Five of them were selected because of the abstract content for complete reading.

Our patient is a 28-year-old man with the following personal background: HIV positive, syphilis, smoker, regular consumer of amphetamines (recreational use).

On October 2017, he went to the emergency room reporting that in the last 72 hours, he had voluntarily injected himself a nonspecific amount of mephedrone in his left forearm. He told us that he usually consumed the drug during sex parties. He first took it intranasally, but because of its tolerance through this method, he started injecting it to achieve a greater effect.

A few hours after drug administration, he began to experience edema, a rash, and pain in the left thumb tip. He also referred that at the time of the injection, he felt paraesthesia in the radial nerve territory of his left forearm, distal to the puncture site and associated with auditive and visual hallucinations. The next day he experienced progressive worsening of the symptoms on the thumb which actually turned violet in color at the fingertip.

**Fig. 1.**
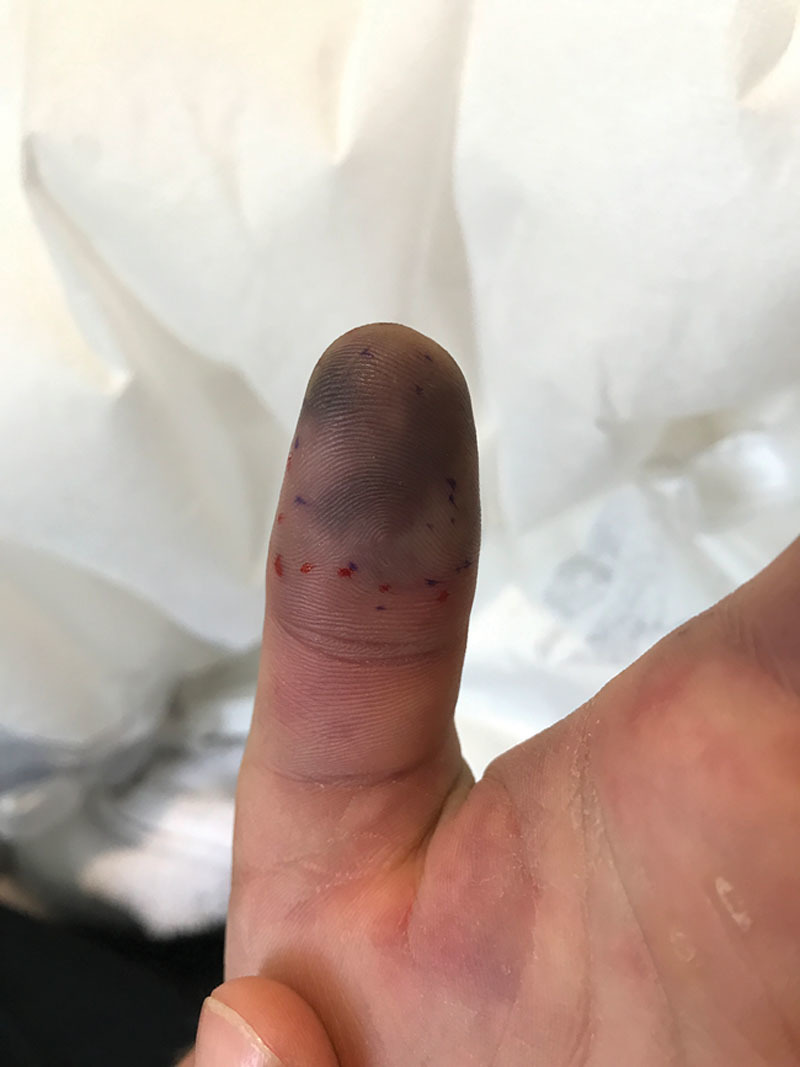
Day 1. The violet color area was marked.

In the physical examination, he presented mild edema confined to the left hand, with pallor at the thenar eminence and cold and violet color areas in the thumb fingertip, which also had hypoesthesia in its ulnar side (Fig. 1). He was able to perform full-range painless extension but he could not do complete flexion because of the edema. The other fingers had good distal capillary refill. We identified the puncture site in the volar side of the distal third in his left forearm, above the radial artery territory.

A routine blood test was carried out without pathological results. We performed an ultrasonography identifying edema and patch necrosis in the thenar muscles. An echocardiogram was not performed because any chest pain nor other cardiac symptoms were found.

We administrated him 10 mg of nifedipine in the emergency room. The patient asked for voluntary discharge against the medical criteria and decided to come back the next day. When he was admitted, we observed further progression of the cyanosis including the thumb fingertip and the radial side of the index.

During the hospitalization, he was treated^6^ with 10 mg of nifedipine per oral every 8 hours, 1 g of amoxicillin-clavulanic acid intravenously (IV) every 8 hours (nontreated HIV), 40 mg of enoxaparin subcutaneously every 24 hours, 2 g of metamizole IV every 8 hours, and 1 g of acetaminophen IV every 8 hours. Additional care included the following: smoking abstinence, extremity rest, raised left arm, and heating blanket (36–40°C) during the whole day.

During the inhospital stay, the patient was examined by Infectious Disease team to initiate treatment of HIV.

## RESULTS

**Fig. 2.**
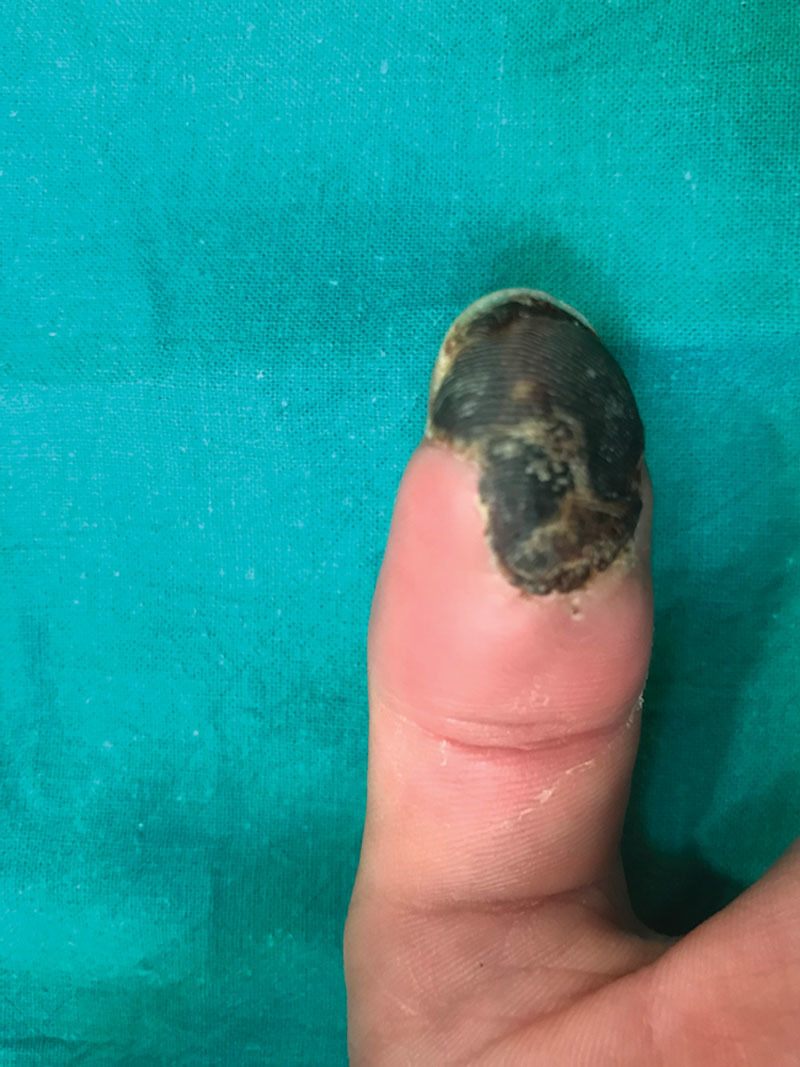
Day 9. Necrosis limited to the thumb fingertip.

During the hospitalization period, we observed sustained cyanosis within the thumb tip with full resolution of the remaining areas. No skin necrosis appeared at the injection site in the forearm. The reason why necrosis limited only to this area remains unclear. By day 9, a well-limited area of necrosis was established (Fig. 2). No bone necrosis was objectified in the x-ray results at any time.

Because of the semiology of the injuries, we supposed that the patient incidentally administrated himself the drug either in the proximity or intra-arterially to his radial artery. Different mechanism could explain the necrosis, such as vasospasm, drug embolism, thrombosis, or arteritis. Unknown underlying previous conditions such as connective tissue diseases might have contributed as well.^7^

**Fig. 3.**
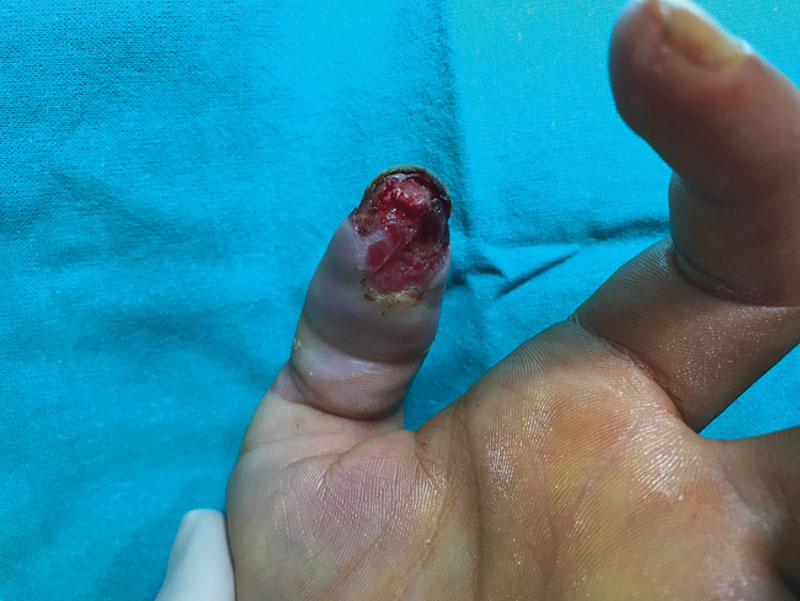
Day 12. Defect after necrosectomy.

**Fig. 4.**
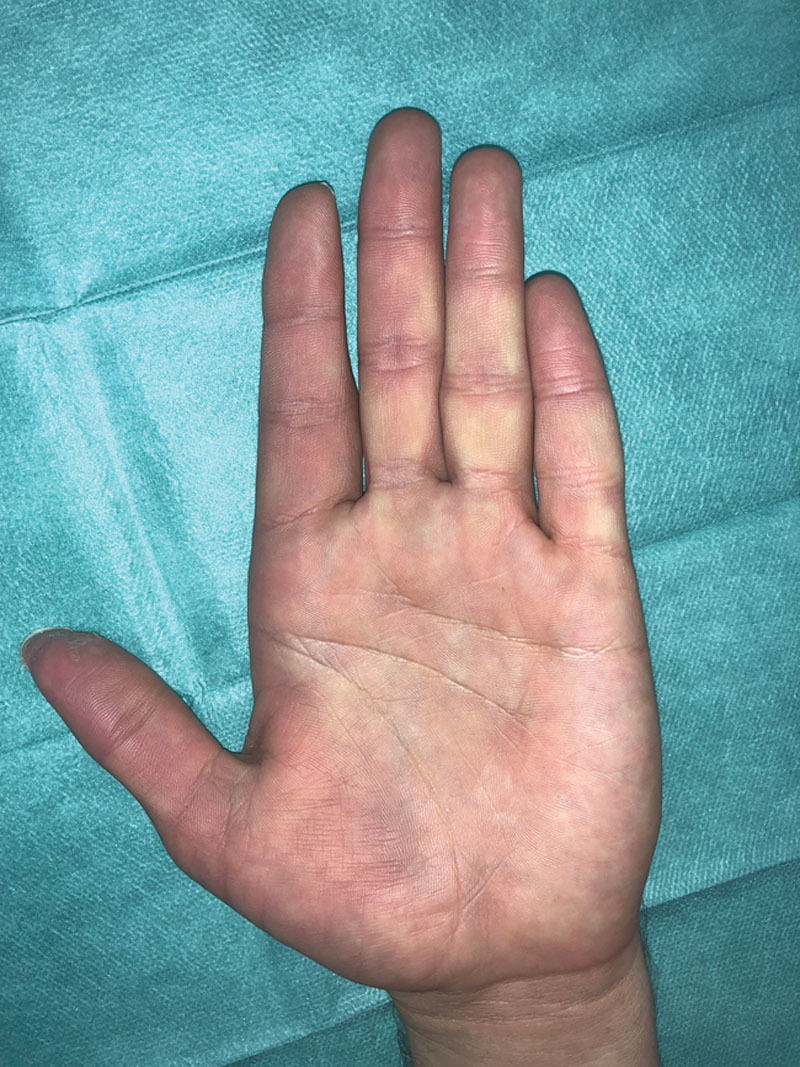
Three weeks after necrosectomy—completely healed.

The patient was managed conservatively in our clinics. We performed a superficial necrosectomy with a subsequent skin and subcutaneous tissue defect of about 4 cm wide (Fig. 3). No bone exposure appeared. Wound care included Linitul (Alfasigma SL, Spain) and gel iodized antiseptics every other day. The defect was completely healed per secundam in 3 weeks achieving normal function and sensitivity of the pulp (Fig. 4). Surgical management was dismissed because of patient characteristics. A possible shortcoming of our treatment is that healing per secundam does not ensure best sensitivity.

**Fig. 5.**
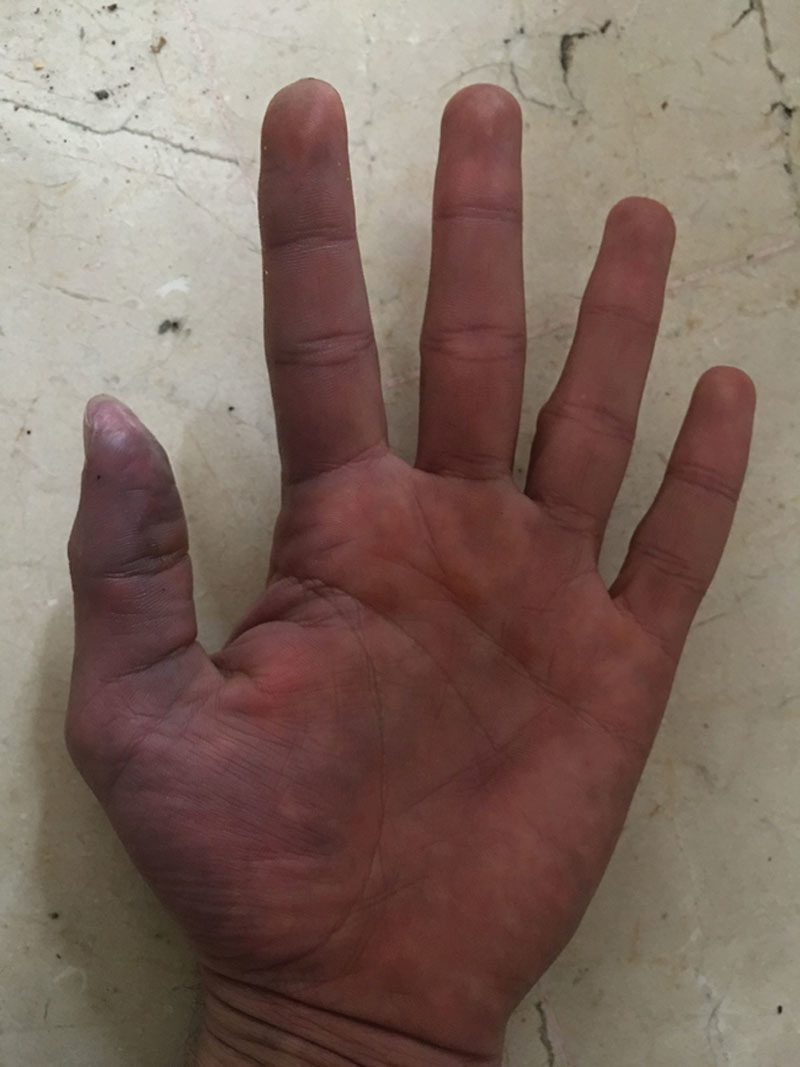
Long-term Raynaud’s phenomenon.

As a single long-term physical damage, the patient refers symptoms consistent with secondary Raynaud’s phenomenon related to abrupt temperature changes (Fig. 5). This can be explained by sympathetic hyperactivity due to mephedrone’s chemical toxicity in the arterial wall, similarly to what happens as a complication of some chemotherapics.^8^ Perivascular injection of a calcium channel blocker or local anesthetic as a chemical sympathectomy will be considered if the symptoms prolong in time.

## CONCLUSIONS

- No local complications have been reported yet after mephedrone intravascular administration.
- A potential side effect is the appearance of distal fingertip necrosis.
- The mechanisms producing these symptoms remain unclear.
